# Early outcomes after carotid angioplasty with stenting performed by neurologists

**DOI:** 10.4103/0972-2327.70883

**Published:** 2010

**Authors:** Lokesh Bathala, Fenglei Zhu, Minmin Ma, Yuping Ma, Gelin Xu, Xinfeng Liu

**Affiliations:** 1 Department of Neurology, Jinling Hospital, Nanjing University School of Medicine, Nanjing, China; 2 Department of Neurology, Narayana Medical College and Hospital, Nellore, India

**Keywords:** Carotid angioplasty and stenting, complications, stroke

## Abstract

**Aims:**

To evaluate the results of carotid artery angioplasty and stenting (CAS) in treating extracranial carotid artery stenosis performed by neurologists in our center and compare the results with other large published series.

**Materials and Methods:**

Data for all patients who underwent CAS from January 2003 through November 2007, was retrieved from the Nanjing Stroke Registry. Perioperative and post-procedural complications within 30 days following stenting were analyzed and compared with that from other series. A total number of 75 patients were enrolled, with a mean age of 65.9 ± 8.8 years, and 64 (85.3%) of them were male.

**Results:**

Procedural success was achieved in 74 patients (98.7%). Pre-treatment stenosis was 73.8 ± 14.9 and post-treatment residual stenosis was less than 10%. Thirty-four patients (45.3%) had bilateral carotid artery disease and seven (9.3%) had tandem stenosis. The neurological complication rate was 3.9% (one major and two minor strokes). Bradycardia in four (5.3%) and hypotension in 13 (17.3%) were observed during procedures. Using the Fischer’s exact t test, the complication rate compared with the large published series did not reveal any statistically significant difference (*P* > 0.05).

**Conclusions:**

We conclude that neurologists, with adequate training, can develop and add this technical skill to the existing cognitive skill of vascular neurology and safely perform stenting.

## Introduction

Stroke is the most devastating consequence of vascular disease causing serious long-term disability and placing an extremely high medical and financial burden on the patient. It is the second leading cause of death and one of the most common causes of disability in adults.[1]

In China, the incidence of stroke is higher than that of coronary artery disease,[2] and each year, more than one million residents die from stroke across the country.[3]

Atherosclerotic disease at the carotid bifurcation has been seen to be responsible for greater than 20 to 25% of all strokes.[4] In the Nanjing Stroke Registry from main land China atherothrombotic strokes accounted for 27.1%.[5]

After the randomized clinical studies of the North American Symptomatic Carotid Endarterectomy Trial (NASCET) and Asymptomatic Carotid Atherosclerosis Study (ACAS), Carotid Endarterectomy (CEA) has been proved to be beneficial in reducing recurrent strokes in symptomatic and asymptomatic patients with significant carotid artery stenosis.[6,7] Endovascular stent placement for carotid artery disease is evolving from its initial controversial position to that of a reasonable alternative to CEA. Single Center experiences with carotid angioplasty and stenting (CAS) across the globe suggest good, immediate, and intermediate results and a low rate of complications.[8,9] Most of the interventions are preformed either by cardiologists, interventional radiologists or vascular surgeons. In this article, we as neurologists, present our experiences of performing CAS and compare our results with other large published series.

## Materials and Methods

Data for patients who underwent CAS have been retrospectively retrieved from the Nanjing Stroke Registry Program (NSRP), which is an ongoing prospective observational project inaugurated in July 2002.[10] Since the beginning of CAS in January 2003, all the details pertaining to the intervention are included in the Registry. A total number of 75 patients had undergone the procedure from January 2003 to November 2007. Indications for CAS include > 50% stenosis, symptomatic, and > 70% stenosis, asymptomatic. All patients were subjected to neuroimaging, either CT or MRI. Carotid Doppler or CTA (computed tomography angiography) or MRA (magnetic resonance angiography) were the initial screening tools for evaluating carotid artery disease. Patients suspected to have carotid stenosis from the initial evaluation were selected for digital subtraction angiography (DSA).

DSA was the gold standard for confirmation of stenosis. Patients were treated with 100 mg aspirin OD, combined with 75 mg Clopidogrel bisulfate OD for five days, before CAS. At discharge patients were advised to continue Clopidrogel for a period of four months and Aspirin life long.

### Definitions used in this study

Transient ischemic attack (TIA) — temporary focal cerebral or retinal deficits that resolved within 24 hours. Minor stroke — a new neurological defi cit that persisted for a period of more than 24 hours, MRS < 3 (MRS-Modified Rankin Scale). Major stroke — a new neurological deficit that persisted beyond 24 hours, disabling, MRS > 3[11].

Severe bradycardia was defined as a heart rate of < 40 beats/min. Hypotension was defined as systolic blood pressure of < 90 mm Hg. Myocardial infarction was defined as the development of a new Q wave on the ECG and / or an increase in creatine kinase (CK) to at least twice the upper limit of normal, associated with elevated levels of CK-MB isoenzymes. Hyperperfusion syndrome was defined as a sudden onset of headache, seizures, confusion, neurological deficit, and high blood pressure (systolic blood pressure 150 mmHg and / or diastolic blood pressure 90 mmHg) following the CAS.

### Carotid angioplasty and stenting

Carotid angioplasty and stenting (CAS) was started in the year 2003, at the Jinling Hospital Department of Neurology, PR China. A team of four neurologists trained in cerebral angiography and CAS performed the procedure once a week. The decision to stent the carotid stenosis was based on DSA (the NASECT method was used to measure the percentage of stenosis)

Carotid artery angioplasty and stenting was performed in the operating room utilizing a digital fluoroscopic imaging system (Advantx LCAz, GE Medical System, Europe) under local anesthesia. Entry access was achieved by placing a 5F introducer sheath in the femoral artery using the Seldinger technique. After selective DSA, the introducer sheath was replaced with an 8F sheath. An 8F guiding catheter (Cordis, Miami, FL, USA) was then placed in the distal common carotid artery. A filterable cerebral protection device, AngioGuard (Cordis, Miami, FL, USA), EPI Filterwire EX (Boston Scientific, USA) or Spider FxEV3 (Minnesota, USA), was used. Protection devices were used in all patients. An over-the-wire angioplasty balloon (Boston Scientific, Maple Grove, MN, USA) was used to pre-dilate stenosis with a pressure of 6 – 8 atmospheres if indicated. At the time of balloon angioplasty, 0.6 mg IV atropine was routinely given to counter any significant bradycardia.

A self-expanding carotid stent, either Wallstent (Boston Scientific, USA), Precise (Cordis, Miami, FL, USA), or Protégé EV3 (North Plymouth, Minnesota USA) was deployed across the stenosis. Post-stenting balloon angioplasty was performed depending on the appearance of the completed angiogram. Clinical success was defined as maximal residual stenosis of less than 30%. Patients were shifted to regular neurological ward if post-procedure hemodynamic parameters and neurological examination were normal. In cases of any abnormality being monitored, patients were sent to the Neurointensive Care Unit.

## Results

A total of 75 patients had undergone CAS since 2003. Males were 64 and females 11 in number. Hypertension was an important risk factor (70%) in our cohort. Other details are mentioned in Table 1. All the patients who presented to us were symptomatic. The left carotid was more affected than the right one. The clinical picture at presentation was TIA 40%, minor stroke 41%, and major stroke 18%.

**Table 1:** Demographic and risk factor characteristics

	Numbers (%)
Male	64 (85.3)
Female	11 (14.7)
Age range (years)	42-80
Mean (SD)	65.9 (8.77)
Hypertension	53 (70.7)
Smoking	25 (33.3)
Diabetes mellitus	18 (24)
Alcohol	20 (26.7)
Dyslipidemia	33 (44)

Mean carotid artery stenosis was 73.8 ± 14. Other angiographic features are mentioned in Table 2. Stenting was performed in 98% of the cases, except in one patient. He was a 62-year-old male who presented with TIA, and had 70% stenosis of the right internal carotid artery (RICA). During the procedure, when the catheter reached the right common carotid artery, the patient developed a generalized tonic–clonic convulsion and postictally had left hemiparesis. Diffusion weighted imaging (DWI) revealed a new infarct in the right lateral temporal lobe. His symptoms improved over the next two weeks with medical management, and at discharge his MRS was 3. Two patients developed minor strokes. One was a 56-year-old female who had central retinal artery occlusion (CRAO) of the right eye post stenting and over three weeks her vision improved, but not to the baseline MRS of 1. The second patient was a male who developed behavioral disturbances lasting for three days after stenting and an MRI showed a new infarct in the anterior thalamus. Within a week his symptoms resolved and at discharge his MRS was 2.

**Table 2:** Angiographic analysis

	Numbers (%)
Mean stenosis	73.8 ± 14.9
Range	50% - 99%
Significant stenosis on right	30 (40)
Significant stenosis on left	45 (60)
Tandem stenosis	7 (9.3)
Bilateral carotid artery disease	34 (45.3)

Despite routine prophylactic atropine before stent placement, hemodynamic disturbances were encountered in 17 patients. Four (5.3%) had bradycardia and 13 patients (17.3%) had hypotension. Ten had transient hypotension lasting for few minutes and in another three it lasted for six to ten hours, needing dopamine infusion.

Post stenting residual stenosis was less than 10%.

Complications were compared with a large series of more than 500 enrolled patients. One was a single center experience and the other three were multicenter data. Results of the Fishers’ exact t test with p value are mentioned in Table 3.

**Table 3:** Comparison of complications with other series (*P* value derived by Fischer’s exact test)

Author	Year	Total no patients	Total no of complications	*P* value
Michael H Wholey	2000	4757	375	0.279
Zahn	2004	668	14	0.241
Wolfram Theiss	2004	3270	328	0.114
Kirsteen R Burton	2005	2992	78	0.449
Lokesh Bbathala	2009	75	3	

## Discussion

We think this is the first reported series performed exclusively by neurologists. In our cohort all patients were symptomatic, probably being a tertiary hospital and specializing in endovascular therapy, most of the patients are referred from other hospitals.

Among the risk factor analysis in our small cohort, hypertension was a major risk factor present in 70% of our patients. This was comparable to the epidemiological studies done by He,[12] where the authors concluded that prevalence of hypertension was strongly associated with the risk of stroke and the geographical variation in stroke incidence and mortality was due mainly to differences in the prevalence of hypertension.

In the patient who developed a major stroke the event happened before stenting, following a seizure; however, the exact etiology could not be identified. In the other two patients, as the events occurred immediately after the procedure we assumed it should be due to emboli dislodgement, despite using a protection device. The composite complication rates were comparable to several other large published series.[13–16]

Endovascular interventions to reopen the arteries and salvage the ischemic brain are promising. Intra-arterial (IA) administration of thrombolytic agents, mechanical removal of thrombus, angioplasty, and stenting are some of the endovascular procedures that are going to revolutionize stroke treatment.[17] Asian countries that experience stroke or transient ischemic attack (TIA) have comparatively more intra-cranial than extra-cranial stenosis, compared to the Western world, which may reflect the need for endovascular interventions if maximum medical therapy has failed.[18–20]

Although carotid endarterectomy is still the gold standard for the management of symptomatic carotid stenosis, studies have shown that carotid artery stenting with the use of an emboli- protection device is not inferior and is also favorable in the high-risk subset of patients with severe coronary, pulmonary, or renal disease.[21,22]

Endovascular stenting is being performed across several hospitals; the procedure being performed by interventional radiologists, neurosurgeons, vascular surgeons, and neurologists. A Position Statement of the American Society of Interventional and Therapeutic Neuroradiology, Society of Interventional Radiology, and the American Society of Neuroradiology concludes that sufficient evidence now exists to recommend that intracranial angioplasty with or without stenting must be offered to symptomatic patients with intracranial stenosis, who have failed medical therapy.[23]

Johnston[24] analyzed the issue of who should be allowed entry into carotid arteries. There is no doubt that interventional cardiologists have an enormous experience in endovascular skills for a decade, yet the challenge posed by the cerebral vasculature is distinct. As mentioned by Johnston, selecting the patients for endovascular treatment and managing complications of cervicocerebral interventions requires knowledge and experience with vascular neurology. In their study, Wolfram Theiss[15] observed that the complication rates were higher in the group seen by neurologists before and after stenting, as compared to those in other groups observed by interventionalists, which highlights the point that a careful neurological examination is very important to look for subtle deficits. Any technical skill has a learning curve and CAS is no exception.[25]

In our center, CAS is performed in the Cardiac Catheter Laboratory and the cardiologists have been supportive colleagues contributing to our learning curve. Neurologists, who already have an in-depth cognitive knowledge of the neurovascular anatomy, pathophysiology, and pathology are better equipped to enter the carotids, with adequate technical training.

## Conclusions

Vascular Neurologists who already have clinical cognitive skills related to cerebrovascular disorders, with adequate technical training, can safely perform the endovascular procedure and offer comprehensive care to stroke patients.
